# Supplementary material for: Pathophysiological Significance of Neutrophilic Transfer RNA-Derived Small RNAs in Asymptomatic Moyamoya Disease
Source: Cells. 2021 May 1;10(5):1086. doi: 10.3390/cells10051086 (PMC8147334; doi:10.3390/cells10051086)

2D Structure	Local AU	Position

```
1114                          1141
                     8mer-1a
5'-taACAAAAT-ATCAATACTTAACGGAAAa-3' utr
    ||   ||  |:|| |||  : |||||||
3'-uuUGCACUAUUGGUGAUGUGAUGCCUUUg-5' tRF
             16151413     7 6 5 4 3 2
           3' Pairing        Seed
```

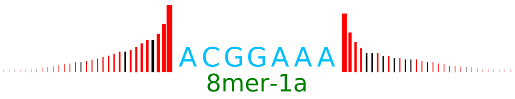

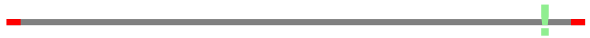

Supplement: Supplementary file 1 [file cells-10-01086-s001.zip › cells-1149161 3rd supplementary materials/Data Supplements/Data Supplement V.zip!/Data supplement 6-mRNAs targets of tsRNAs/tsRNA-Val-AAC-017/Diagrams/tRF-Val-AAC-017_vs_ENST00000610581(SPRY1).pdf]
